# Unspliced Precursors of NMD-Sensitive β-Globin Transcripts Exhibit Decreased Steady-State Levels in Erythroid Cells

**DOI:** 10.1371/journal.pone.0038505

**Published:** 2012-06-04

**Authors:** Ana Morgado, Fátima Almeida, Alexandre Teixeira, Ana Luísa Silva, Luísa Romão

**Affiliations:** 1 Departamento de Genética, Instituto Nacional de Saúde Dr. Ricardo Jorge, Lisboa, Portugal; 2 Centro de Investigação em Genética Molecular Humana, Faculdade de Ciências Médicas, Universidade Nova de Lisboa, Lisboa, Portugal; 3 BioFIG–Center for Biodiversity, Functional and Integrative Genomics, Faculdade de Ciências, Universidade de Lisboa, Lisboa, Portugal; German Cancer Research Center, Germany

## Abstract

Nonsense-mediated mRNA decay (NMD) is a quality control mechanism that detects and rapidly degrades mRNAs carrying premature translation-termination codons (PTCs). Mammalian NMD depends on both splicing and translation, and requires recognition of the premature stop codon by the cytoplasmic ribosomes. Surprisingly, some published data have suggested that nonsense codons may also affect the nuclear metabolism of the nonsense-mutated transcripts. To determine if nonsense codons could influence nuclear events, we have directly assessed the steady-state levels of the unspliced transcripts of wild-type and PTC-containing human β-globin genes stably transfected in mouse erythroleukemia (MEL) cells, after erythroid differentiation induction, or in HeLa cells. Our analyses by ribonuclease protection assays and reverse transcription-coupled quantitative PCR show that β-globin pre-mRNAs carrying NMD-competent PTCs, but not those containing a NMD-resistant PTC, exhibit a significant decrease in their steady-state levels relatively to the wild-type or to a missense-mutated β-globin pre-mRNA. On the contrary, in HeLa cells, human β-globin pre-mRNAs carrying NMD-competent PTCs accumulate at normal levels. Functional analyses of these pre-mRNAs in MEL cells demonstrate that their low steady-state levels do not reflect significantly lower pre-mRNA stabilities when compared to the normal control. Furthermore, our results also provide evidence that the relative splicing efficiencies of intron 1 and 2 are unaffected. This set of data highlights potential nuclear pathways that might be promoter- and/or cell line-specific, which recognize the NMD-sensitive transcripts as abnormal. These specialized nuclear pathway(s) may be superimposed on the general NMD mechanism.

## Introduction

Nonsense-mediated mRNA decay (NMD) is a cellular surveillance mechanism that selectively identifies and rapidly degrades mRNAs containing premature translation-termination codons (PTCs). Therefore, by downregulating mRNAs bearing nonsense codons, NMD prevents the synthesis of C-terminally truncated proteins potentially toxic for the cell [1], [2]. As about one third of all known disease-causing mutations originate a nonsense codon, NMD may function as a significant modulator of genetic disease phenotypes in humans [1]–[3]. Moreover, many physiological mRNAs have been recently described as NMD substrates, suggesting an additional role for NMD as a posttranscriptional regulator of gene expression [3]–[5].

NMD has been extensively studied for decades in yeast, worms, fruit fly, plants and mammals, and several models have been proposed depicting different aspects of the NMD machinery, such as nonsense codon recognition or subcellular localization, amongst others [6]–[9]. In mammalian cells, NMD depends on the interaction of the termination complex with a multi-component exon-junction complex (EJC) [6]–[9]. The EJC is deposited 20–24 nucleotides (nts) upstream of each exon-exon junction during splicing [10]. According to the present model for mammalian NMD, the EJC, or a critical subset of EJC components, remains associated with the mRNA during its transport to the cytoplasm. Translating ribosomes subsequently displace EJCs from the open reading frame during the first (‘pioneer’) round of translation [11], [12]. However, if an mRNA contains a PTC located more than 50–54 nts upstream the last exon-exon junction, the ribosome will fail to displace distal EJC(s). In this case, when the ribosome reaches the PTC, the translation release factors eRF1 and eRF3 at the PTC interact in *cis* with the retained EJC(s) via a multiprotein bridge [13]. Of central importance in this process is the interaction of UPF1 and SMG1 with the terminating complex and with the UPF2/UPF3 components of the retained EJC(s) [13]. This bridging interaction triggers the mRNA for rapid decay (i.e., NMD) of the PTC-containing mRNA.

Despite the translational-dependence of NMD, most mRNAs harbouring PTCs sh–ow reduced steady-state levels not only in the cytoplasm, but also in the nuclear fraction of mammalian cells [14]–[19]. These apparently conflicting data are explained by the model postulating that mRNAs are read by ribosomes while they are exported to the cytoplasm, which prompts the degradation of nonsense-containing mRNAs still associated with the nucleus [12].

Whether mammalian cells can recognize the presence of a nonsense codon before mRNA processing and export from the nucleus has remained a topic of discussion [20]. For instance, some evidences account for a link between premature translation-termination events and nuclear events, or for translation within the nucleus [21]–[23]. Regarding the nuclear metabolism of nonsense transcripts, several authors observed that the presence of a nonsense codon could alter the pre-mRNA splicing pattern. This effect was attributed to the disruption of exonic splicing enhancers or RNA secondary structure forced by the PTC [24]–[27]. Nonsense codons have also been reported to inhibit pre-mRNA splicing in an open reading frame-dependent manner [28]–[30]. Recently it has been described that the immunoglobulin-μ unspliced transcripts containing nonsense codons are specifically retained at the transcription site. This RNA retention is dependent on two essential NMD factors, UPF1 and SMG6, and indicates that a mechanism for regulation of PTC-bearing transcripts might occur at the site of transcription [31].

In the present study, we tested whether the nuclear metabolism of nonsense-mutated transcripts is altered in mammalian cells. We therefore examined the steady-state levels of normal and nonsense-mutated human β-globin pre-mRNAs stably expressed in mouse erythroleukemia (MEL) and HeLa cells. Our data revealed that the presence of a NMD-competent PTC specifically affects the abundance of the corresponding β-globin pre-mRNAs in erythroid cells, although not affecting their pre-mRNA half-lives. However, in the non-erythroid cells, reduction of pre-mRNA levels is not observed. Our results therefore underline a specific effect of the NMD-competent PTC on the nuclear metabolism of the corresponding transcripts.

## Results

### Human β-globin pre-mRNAs carrying a nonsense mutation accumulate at low levels

**Figure 1:**
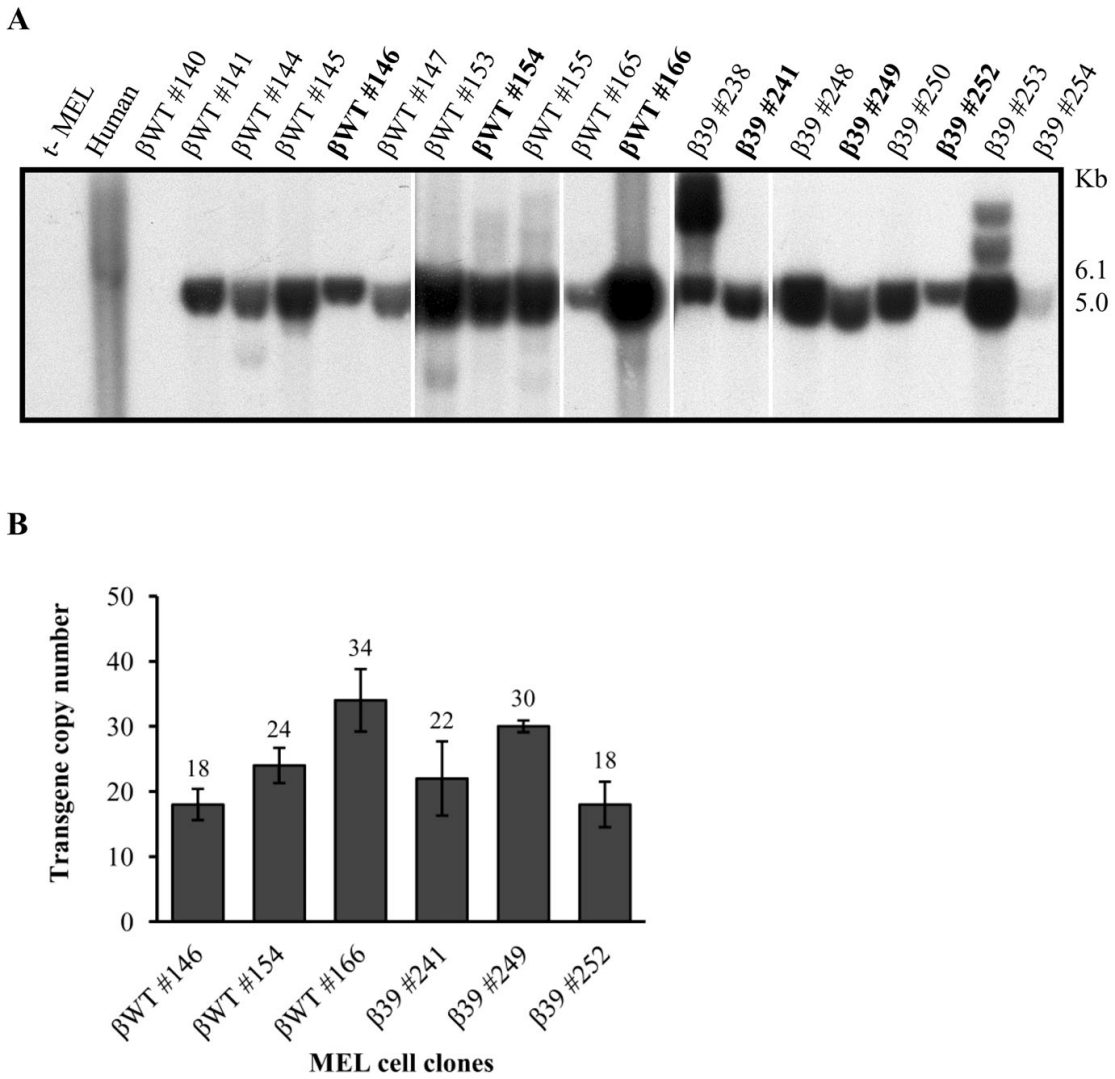
Human β-globin transgene integrity and copy number analysis in stably transfected MEL cell clones. (A) Representative Southern blot analysis of DNA from MEL cell clones stably transfected with wild-type (βWT) or nonsense-mutated (β39; CAG→UAG) human β-globin gene constructs. Genomic DNA was extracted from MEL cells transfected with a β-globin construct as specified above each lane, where each number indicates an independent cell clone. Untransfected MEL (t- MEL) and human genomic DNA were used as negative and positive controls, respectively. DNA was digested with EcoNI plus KpnI enzymes and blots were hybridized with a [α-^32^P]dCTP-labeled probe of the human β-globin gene that recognizes a 5.0 kb fragment integrated in the murine genome or a 6.1 kb fragment in the human genomic DNA. MEL cell clones selected for further analysis are indicated in bold. (B) Transgene copy number for each selected MEL cell clone was determined by quantitative PCR using primers specific for human β-globin gene and the endogenous murine Thy1 gene. Quantification was performed by the relative standard curve method. Chart shows the mean ± standard deviation qPCR data from three independent experiments.

With the aim to investigate if the presence of a nonsense codon in a transcript could affect its nuclear metabolism, in this study, we generated stably transfected MEL cell clones expressing the wild-type human β-globin gene (βWT), or a β-globin gene variant carrying a nonsense mutation at codon 39 (β39), which is a well-characterized β-globin NMD substrate in erythroid as well as in non-erythroid cells [32]–[35]. Each human β-globin gene was cloned into the p158.2 vector, as previously described, where it is expressed under the transcriptional control of the corresponding promoter and the DNase I hypersensitive site 2 of the human locus control region [32]. To select cell line clones for further studies, the integration of the intact human β-globin gene in the murine genome was evaluated by Southern blot analysis (Figure 1A). From the different clones analysed, we have chosen, for further analyses, six independent clones – #146, #154, #166 expressing the βWT gene, and #241, #249 and #252 expressing the β39 gene. The chosen cell clones show the same pattern of integration and different copy number of integrated transgenes. In these selected clones, accurate evaluation of the human β-globin transgene copy number was performed by quantitative PCR using the endogenous diploid thymus cell antigen (Thy1) gene as a copy number reference. Results have shown that for the three βWT independent selected clones, the corresponding transgene copy number is 18±2, 24±3 and 34±5, whereas in the β39 cell lines, the transgene copy number is 22±6, 30±1 and 18±2 (Figure 1B). This selection allowed us to perform further gene expression analyses in pairs of βWT and β39 clones matched for transgene copy number.

**Figure 2:**
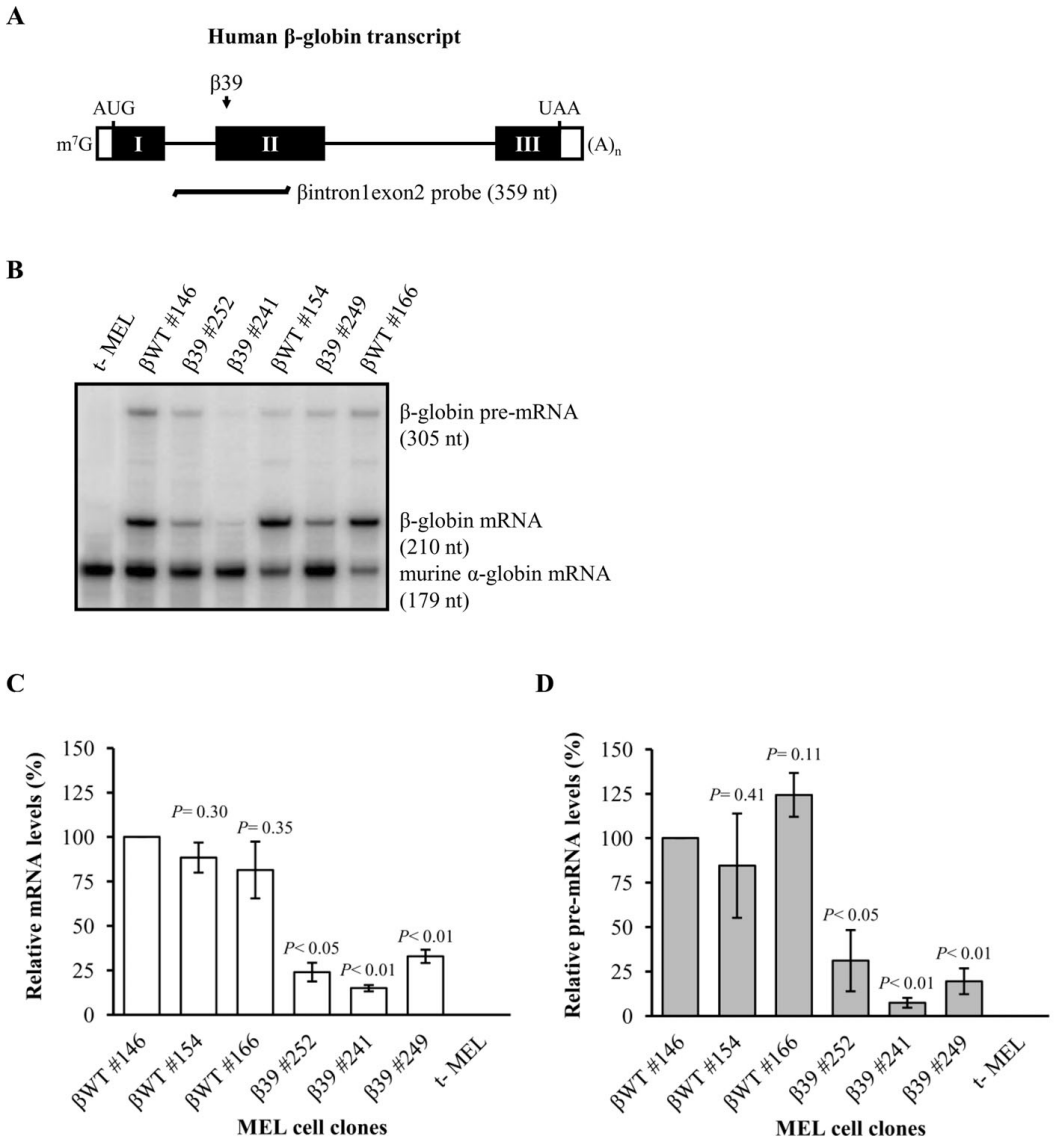
Human β-globin pre-mRNAs carrying a nonsense mutation accumulate at low levels in MEL cells. (A) Schematic representation of the test human β-globin constructs stably expressed in MEL cell lines. The closed and open rectangles and lines depict exons, untranslated sequences and introns, respectively. The vertical small arrow represents the position of the nonsense mutation (CAG→UAG) at codon 39 (β39). Position of initiation (AUG) and termination (UAA) codons, as well as cap structure (m^7^G) and poly(A) tail [(A)_n_] are also represented. Localization and length in nucleotides (nt) of the probe comprising intron 1-exon 2 sequences (βintron1exon2 probe) for detection and quantification of the human β-globin RNA by ribonuclease protection assays (RPA) is shown below the diagram. (B) MEL cells were stably transfected with a test human β-globin construct as specified in each lane, where each number indicates an independent MEL cell line. After erythroid differentiation induction, steady-state total RNA from either transfected or untransfected (t-) MEL cells was isolated and analysed by RPA using specific probes for human β- and mouse α-globin transcripts (see Materials and Methods). The protected bands corresponding to the human β-globin pre-mRNA and mRNA and mouse α-globin mRNA are shown on the right, and the corresponding intensities were quantified by phosphorimaging. The level of mRNA and pre-mRNA from each β-globin allele was normalized to the level of endogenous mouse α-globin in order to control for RNA recovery and erythroid differentiation induction. Normalized values were then calculated as the percentage of wild-type β-globin (βWT) mRNA from cell line #146 (arbitrary defined as 100%). The values exposed on the graphs (C) and (D) are representative of three independent experiments, and are plotted for each construct showing the mean value and standard deviations. Statistical analysis was performed using the Student's *t* test (unpaired, two-tailed).

To assess the effect of the PTC on the nuclear metabolism of the β-globin transcripts, we compared, by ribonuclease protection assays (RPA), the steady-state expression levels of βWT and β39 transcripts in the selected MEL cell lines after induction of erythroid differentiation by dimethyl sulfoxide (DMSO) (see Materials and Methods). Using a ^32^P-labelled riboprobe spanning β-globin intron 1 and exon 2 sequences (Figure 2A), the pre-mRNA as well as the processed mRNA from total RNA were simultaneously detected and quantified (Figure 2B). The hybridization signals of both β-globin spliced and unspliced transcripts from all MEL cell clones were normalized to the murine α-globin mRNA signal produced by the respective riboprobe, and estimated as a percentage of the normalized value for the βWT #146 clone (arbitrarily considered 100%). Our results show that the β39 MEL cell clones exhibit reduced β-globin mRNA levels, in agreement with rapid decay by NMD, as expected [32]–[35] (Figure 2C). Remarkably, all β39 MEL cell clones display a significant 3- to 14-fold reduction in the pre-mRNA steady-state levels relatively to the reference βWT #146 pre-mRNA level, and relatively to the pre-mRNA level from the corresponding βWT clone with equivalent transgene copy number (Figure 2D). These results suggest that the presence of a NMD-sensitive nonsense codon can affect the metabolism of the unspliced β-globin transcripts in MEL cells nuclei, independently of the transgene copy number.

### The low levels of the β39 pre-mRNAs are PTC-specifc

**Figure 3:**
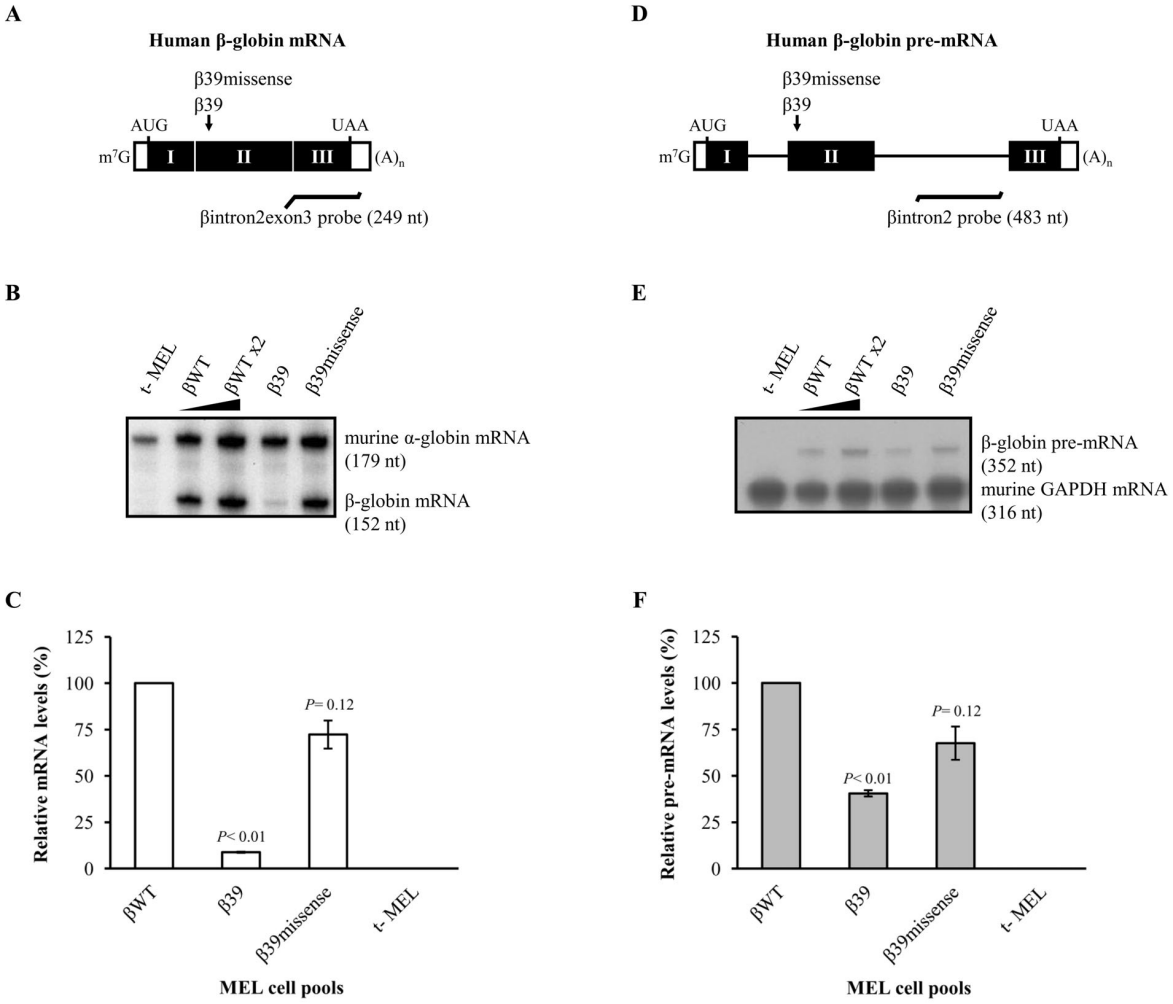
The low levels of the β39 pre-mRNA are not due to the disruption of a regulatory element encompassing codon 39. (A) Schematic representation of the test human β-globin mRNA stably expressed in MEL cell pools. The closed and open rectangles depict exons and untranslated regions, respectively. The vertical small arrow represents the position of the nonsense (CAG→UAG) or missense (CAG→GAG) mutation at codon 39 (β39 and β39missense respectively). Position of initiation (AUG) and termination (UAA) codons, as well as cap structure (m^7^G) and poly(A) tail [(A)_n_] are also represented. Localization and length in nucleotides (nt) of the probe comprising intron 2-exon 3 sequences (βintron2exon3 probe) for detection and quantification of the human β-globin RNA by ribonuclease protection assays (RPA) is shown below the diagram. (B) MEL cells were stably transfected with a test human β-globin construct as specified above each lane. A 2-fold RNA sample (βWT×2) from MEL cells transfected with the βWT gene was also assayed to demonstrate that the experimental RPA was carried out in probe excess. After erythroid differentiation induction, steady-state total RNA from either transfected or untransfected (t-) MEL cells was isolated and analysed by RPA using specific probes for human β- and mouse α-globin mRNAs (see Materials and Methods). The protected bands corresponding to the human β-globin and mouse α-globin mRNAs are shown on the right, and the corresponding intensities were quantified by phosphorimaging. The level of mRNA from each β-globin allele was normalized to the level of endogenous mouse α-globin in order to control for RNA recovery and erythroid differentiation induction. Normalized values were then calculated as the percentage of wild-type β-globin mRNA. (C) The percentage mRNA values were plotted for each construct, and standard deviations from three independent experiments are shown. Statistical analysis was performed using Student's *t* test (unpaired, two-tailed). (D) Schematic representation of the test human β-globin pre-mRNA stably expressed in MEL cell pools as in (*A*). Localization and length in nucleotides (nt) of the probe comprising part of intron 2 (βintron2 probe) for detection and quantification of the human β-globin pre-mRNA by RPA is shown below the diagram. (E) After erythroid differentiation induction, steady-state total RNA from either transfected or untransfected (t-) MEL cells was isolated and analysed by RPA using specific probes for human β-globin pre-mRNA and mouse glyceraldehyde 3-phosphate dehydrogenase (GAPDH) mRNA (see Materials and Methods). The corresponding protected bands are shown on the right, and their intensities were quantified by phosphorimaging as in (*B*). (F) The percentage pre-mRNA values were plotted for each construct, and standard deviations from three independent experiments are shown, as in *(C)*.

In order to discard a pleiotropic effect of the β39 nonsense mutation that, for example, could disrupt an exonic splicing regulatory element surrounding codon 39, we generated MEL cell pools stably expressing a β-globin construct bearing a different mutation at codon 39–a missense mutation (β39missense; see Materials and Methods). After erythroid cell differentiation induction, the mRNA levels were determined by RPA, as before, using probes comprising part of the human β-globin intron 2 and exon 3 or murine α-globin mRNA sequences. Results were compared to those of MEL cell pools stably expressing the βWT or β39 genes (Figure 3A–C). Our data show that the β39missense mRNA level accumulates at about 72% of the βWT mRNA, while β39 mRNAs accumulate at about 9% of the normal control (Figure 3C). As expected, these results show that the missense mutation at codon 39 does not significantly affect the corresponding steady-state mRNA accumulation level (*P* = 0.12).

In parallel, β39missense pre-mRNA levels were also quantified by RPA using a probe specific for the second intron (βintron2 probe; Figure 3D), whose intensity was normalized with the murine glyceraldehyde 3-phosphate dehydrogenase (GAPDH) mRNA signal generated by the respective riboprobe protection and compared to the βWT and β39 controls (Figure 3E, F). These analyses revealed that the β39missense pre-mRNA accumulates at about 68% of the βWT pre-mRNA (*P* = 0.12), while β39 unspliced mRNA accumulates at about 40% of the normal (*P*<0.01), showing that, contrary to what occurs with the β39 pre-mRNA, the β39missense pre-mRNA is not significantly decreased. Taken together, these results clearly show that the steady-state decreased levels of β39 pre-mRNA are not due to a pleiotropic effect of the mutation at position 39, but, instead, they seem to be PTC-specific.

### The decreased β-globin pre-mRNA levels are specific for transcripts carrying NMD-sensitive nonsense codons

**Figure 4:**
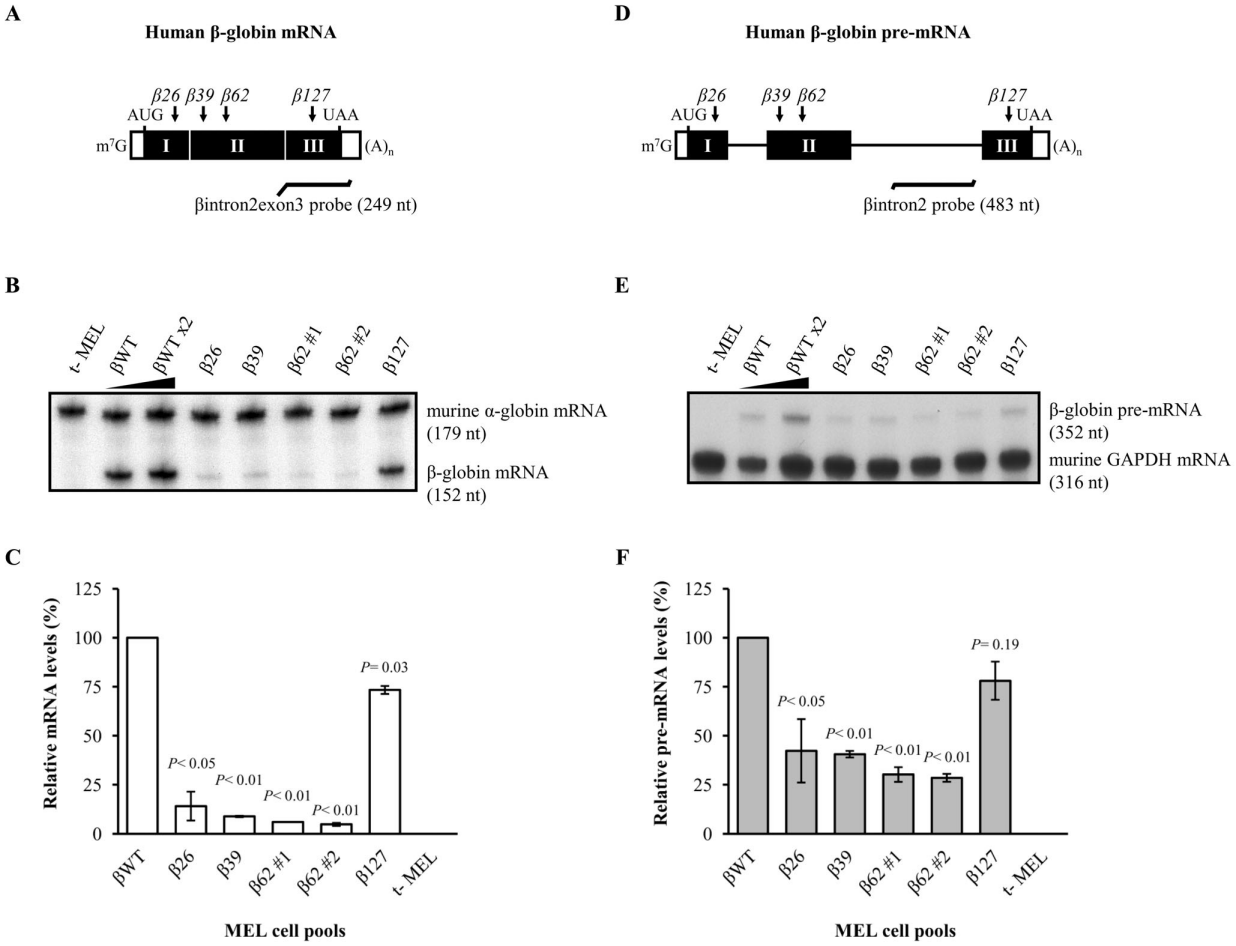
The decreased β-globin pre-mRNA levels are specific for transcripts carrying NMD-competent nonsense mutations. (A) Schematic representation of the test human β-globin mRNA stably expressed in MEL cell pools. The closed and open rectangles depict exons and untranslated regions, respectively. The vertical small arrows represent the position of the nonsense mutations at codon 26 (GAG→UAG; β26), 39 (CAG→UAG; β39), 62 (GCT→UAG; β62) or 127 (CAG→UAG; β127). Position of initiation (AUG) and termination (UAA) codons, as well as cap structure (m^7^G) and poly(A) tail [(A)_n_] are also represented. Localization and length in nucleotides (nt) of the probe comprising intron 2-exon 3 sequences (βintron2exon3 probe) for detection and quantification of the human β-globin RNA by ribonuclease protection assays (RPA) is shown below the diagram. (B) MEL cells were stably transfected with a test human β-globin construct as specified above each lane. A 2-fold RNA sample (βWT×2) from MEL cells transfected with the βWT gene was also assayed to demonstrate that the experimental RPA was carried out in probe excess. After erythroid differentiation induction, steady-state total RNA from either transfected or untransfected (t-) MEL cells was isolated and analysed by RPA using specific probes for human β- and mouse α-globin mRNAs (see Materials and Methods). The protected bands corresponding to the human β-globin and mouse α-globin mRNAs are shown on the right, and the corresponding intensities were quantified by phosphorimaging. The level of mRNA from each β-globin allele was normalized to the level of endogenous mouse α-globin in order to control for RNA recovery and erythroid differentiation induction. Normalized values were then calculated as the percentage of wild-type β-globin mRNA. (C) The percentage mRNA values were plotted for each construct, and standard deviations from three independent experiments are shown. Statistical analysis was performed using Student's *t* test (unpaired, two-tailed). (D) Schematic representation of the test human β-globin pre-mRNA stably expressed in MEL cell pools as in (*A*). Localization and length in nucleotides (nt) of the probe comprising part of intron 2 (βintron2 probe) for detection and quantification of the human β-globin pre-mRNA by RPA is shown below the diagram. (E) After erythroid differentiation induction, steady-state total RNA from either transfected or untransfected (t-) MEL cells was isolated and analysed by RPA using specific probes for human β-globin pre-mRNA and mouse glyceraldehyde 3-phosphate dehydrogenase (GAPDH) mRNA (see Materials and Methods). The corresponding protected bands are shown on the right, and their intensities were quantified by phosphorimaging as in (*B*). (F) The percentage pre-mRNA values were plotted for each construct, and standard deviations from three independent experiments are shown, as in (*C*). Statistical analysis was performed using Student's *t* test (unpaired, two-tailed).

Considering the formerly observed downregulation of unspliced β-globin transcripts carrying a nonsense mutation at codon 39, we next asked whether this effect occurs in other transcripts carrying a different PTC. We thus established two different MEL cell pools stably expressing the human β-globin gene carrying a NMD-sensitive nonsense mutation at codon 26 (exon 1; β26) or at codon 62 (exon 2; β62) [36]. The β26 and β62 mRNAs were previously found to accumulate at reduced steady-state levels when compared to the wild-type β-globin mRNA in erythroid and non-erythroid cells [32], [36] These transcripts are NMD-sensitive because the respective PTCs are located more than 50–54 nts upstream to the 3′-most exon-exon junction and when the ribosome reaches the PTC, the terminating complex can interact with the downstream EJC *via* UPF1 [36], [37]. Regarding β62 MEL cell pools, we were able to isolate two independent MEL cell pools (β62#1 and β62#2). After erythroid cell differentiation induction, the transgene mRNA levels were determined by RPA as before, using probes comprising part of the human β-globin intron 2 and exon 3 or murine α-globin mRNA sequences, and results were compared to those of MEL cell pools stably expressing the βWT or β39 genes (Figure 4A, B). According to our previously published data [36], our results show that β26 and β62 mRNA levels of the corresponding MEL cell pools are strongly downregulated relatively to the βWT mRNA levels, presenting levels similar to those observed in the β39 MEL cell pools, meaning that they are induced to rapid decay, as expected (Figure 4B, C). These results indicate that under our experimental conditions, the cellular NMD machinery is functional.

At these experimental conditions, the pre-mRNA levels of the β26 and β62 MEL cell pools were quantified using a probe specific for the second intron (βintron2 probe; Figure 4D), whose intensity was normalized with the murine GAPDH mRNA signal generated by the respective riboprobe protection (Figure 4E, F), as before. RPA analysis revealed that the β26 and β62 pre-mRNA steady-state levels are at about 40% and 30% of the normal control, respectively. These levels are significantly lower relatively to the βWT pre-mRNA (*P*<0.05 and *P*<0.01, respectively for β26 and β62), being comparable to that of β39 pre-mRNA (Figure 4E, F). These results clearly demonstrate that the reduced nonsense pre-mRNA levels phenotype in MEL cells is independent of the position of the PTC.

Knowing that the reduced nonsense pre-mRNA levels phenotype is PTC-specific, and independent of the PTC position, we next asked if it depends on NMD. Thus, we also established a pool of MEL cells stably expressing the human β-globin gene carrying a nonsense mutation at codon 127 located at the 3′-most exon (β127) that does not induce NMD, as it is located downstream of the 3′-most exon-exon junction [32]–[34], [38]. The mRNA and pre-mRNA levels were quantified as before. Results show that β127 mRNA steady-state levels are at about 73% of the normal control (Figure 4B, C), showing that this transcript is not efficiently degraded by the NMD pathway, as expected [11]. In parallel, β127 pre-mRNA levels were also quantified and compared to those of the normal control. Our data show that β127 pre-mRNA accumulates at about 78% of the βWT pre-mRNA (Figure 4E, F), being this difference not significant (*P* = 0.19). Together, this full set of data shows that the decreased β-globin pre-mRNA levels phenotype is specific for transcripts carrying a NMD-sensitive nonsense codon.

### The presence of an NMD-sensitive nonsense codon does not affect the relative rates of removal of introns 1 and 2 in the human β-globin pre-mRNAs

**Figure 5:**
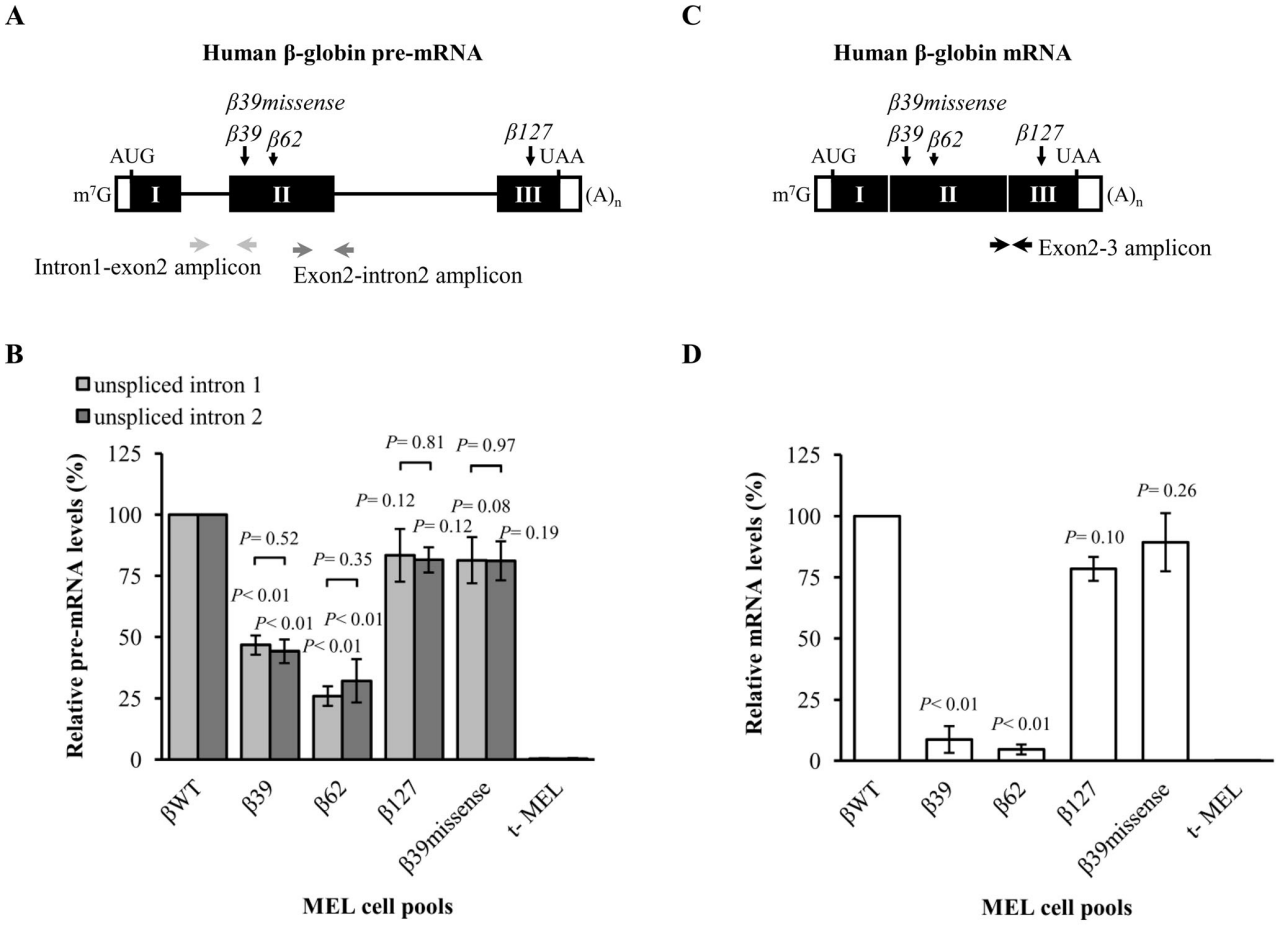
The presence of the nonsense codon equally decreases the abundance of intron 1 *versus* intron 2 containing human β-globin pre-mRNAs. (A) Schematic representation of the human β-globin pre-mRNA, as in Figures 3D and 4D. The two pairs of arrows represent the coordinates of both amplicons obtained in the qPCR reactions: intron1-exon2 and exon2-intron2 amplicons. (B) MEL cells were stably transfected with a test human β-globin construct as specified below the histogram. After erythroid differentiation induction, steady-state total RNA from either transfected or untransfected (t-) MEL cells was isolated and analysed by reverse transcription-coupled quantitative PCR (RT-qPCR), with specific primers for the human β-globin pre-mRNA, as shown in (*A*). For each case, intron 1 and intron 2 containing human β-globin pre-RNAs levels were determined by normalization to the level of murine glyceraldehyde 3-phosphate dehydrogenase (GAPDH) mRNA, using the comparative C_t_ method, and compared to the wild-type control. The percentage pre-mRNA values were plotted for each construct and the histogram shows the mean and standard deviations from three independent experiments. Statistical analysis was performed using Student's *t* test (unpaired, two-tailed). (C) Schematic representation of the studied human β-globin mRNAs as in Figures 3A and 4A. The pair of arrows represents the coordinates of the amplicon obtained in the qPCR reactions: exon2–3 amplicon. (D) Human β-globin mRNA quantification was performed by RT-qPCR as in (*B*), but using specific primers for the human β-globin processed mRNA. Levels of each human β-globin mRNA variant were determined by normalization to the level of murine GAPDH mRNA, using the comparative Ct method, and compared to the wild-type control. The histogram shows the mean and standard deviations from three independent experiments. Statistical analysis was performed as in (*B*).

In order to test to what extent the presence of the nonsense codon affects the relative amount of intron 1 *versus* intron 2 containing β-globin pre-mRNAs, we analysed the β39 and β62 transcripts stably expressed in differentiated MEL cell pools and results were compared to those of the βWT, β127 and β39missense control transcripts. This analysis was carried out by reverse transcription-coupled quantitative PCR (RT-qPCR) assays to specifically quantify the amount of either intron 1 or intron 2 containing human β-globin pre-mRNAs (Figure 5). Thus, pre-mRNA quantification was carried out with two sets of primers specific for the human β-globin intron 1 and intron 2 pre-mRNA sequence, respectively, using a set of primers specific for the murine GAPDH mRNA as an internal control (Figure 5A, B). As a control, RT-qPCR was also performed with a set of specific primers to quantify processed mRNA, to show that, under these experimental conditions, the PTCs at position 39 or 62 are able to induce a strong downregulation of the steady-state levels as expected for mRNAs typically committed to NMD, while levels of mRNA bearing a PTC at the 3′-most exon (β127) are not significantly different from the normal control (Figure 5C, D). The quantitative PCR efficiency for all amplicons was found to be similar and near to 100%. Control reactions using total RNA samples from untransfected MEL cells, confirmed that unspecific amplification of the murine β-globin transcripts was negligible. In agreement with the previously obtained RPA data, RT-qPCR analysis of the intron 2-containing pre-mRNA steady-state levels shows a significant 2.3 to 3.8-fold reduction of the β39 and β62 unspliced RNAs relatively to the βWT pre-mRNA (*P*<0.01) (Figure 5B). On the other hand, β127 and β39missense unspliced transcripts exhibit similar levels, which are not significantly different from the normal control (*P* = 0.12 and *P* = 0.08, respectively). Additionally, in each case, both β-globin intron 1 and intron 2 containing pre-mRNAs yielded very similar expression levels (*P*>0.05). Therefore, the presence of the NMD-sensitive nonsense codons does not differentially affect the rates of removal of intron 1 and 2, and, thus, splicing efficiency in transcripts bearing NMD-competent nonsense codons seems to be normal.

### The reduced steady-state pre-mRNA level of NMD-sensitive transcripts does not reflect differential decay rates

**Figure 6:**
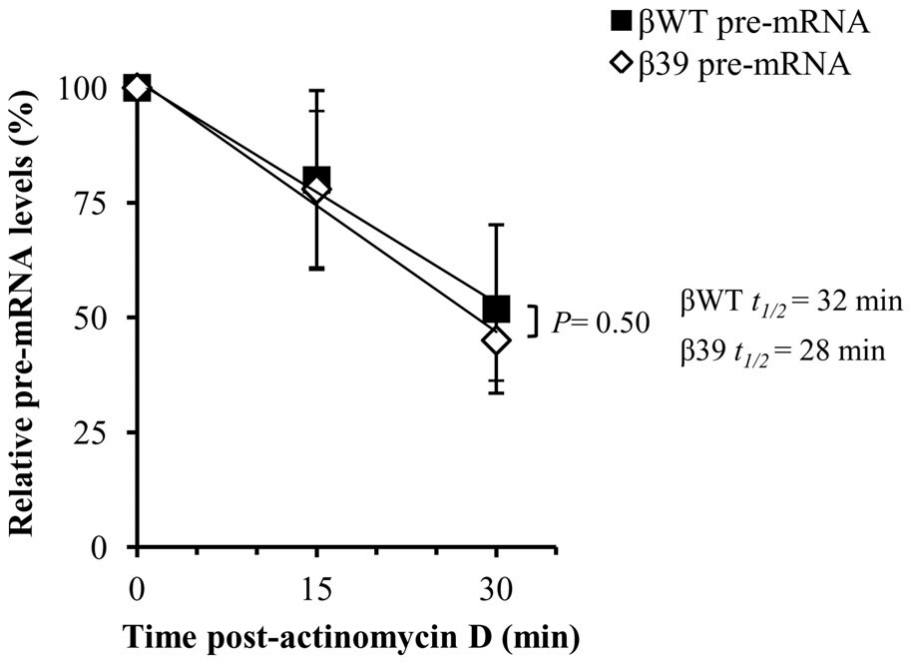
The half-life of a pre-mRNA carrying an NMD-sensitive PTC is not significantly different from that of the wild-type control pre-mRNA. To determine the pre-mRNA decay kinetics, erythroid differentiated MEL cell pools stably expressing the βWT or the β39 transgenes were incubated with 5 μg/mL of actinomycin D. Total RNA was extracted at the indicated times (0, 15 and 30 min) after actinomycin treatment. Relative pre-mRNA levels were measured by RT-qPCR, as described before. For that, the amount of human β-globin pre-mRNA was normalized against the amount of murine GAPDH mRNA and then re-normalized to the initial time point value (time 0 = 100%). Each point represents the mean and standard error mean from three independent experiments. Linear regression analysis was performed by standard techniques and the difference between slopes was assessed by Student's *t* test (two-tailed). The half-lives (t_1/2_) of the pre-mRNAs are indicated.

As the steady-state level of any unspliced transcript depends on the balance between the rate of its transcription and splicing and/or degradation, we next asked if the low steady-state pre-mRNA levels of the NMD-sensitive transcripts indeed reflect increased decay rates rather than changes at the transcriptional level. Thus, we determined the decay kinetics of the β39 pre-mRNA relatively to that of the wild-type control pre-mRNA stably expressed in MEL cells. For this purpose, we treated the erythroid differentiated βWT and β39 MEL cell pools with actinomycin D to inhibit RNA synthesis. Total RNA was isolated at three time points after actinomycin D treatment. As before, the amount of unspliced human β-globin transcripts was determined by RT-qPCR (Figure 6). Results show that the βWT pre-mRNA has an average half-life of 32 min. The presence of the PTC at position 39 does not significantly accelerate the decay of the reporter pre-mRNA as it results in a half-life of 28 min (*P* = 0.50) (Figure 6). Although βWT and β39 pre-mRNAs are not similarly abundant, the β39 turns at similar rates of those of βWT pre-mRNA. These similar values of half-lives are in agreement with a previous study by Lim et al. [39]. Our results suggest that low steady-state pre-mRNA levels of NMD-sensitive transcripts might be due to changes at the transcriptional level.

### The NMD-competent PTC effect on β-globin pre-mRNA abundance exhibits promoter and/or cell line specificity

**Figure 7:**
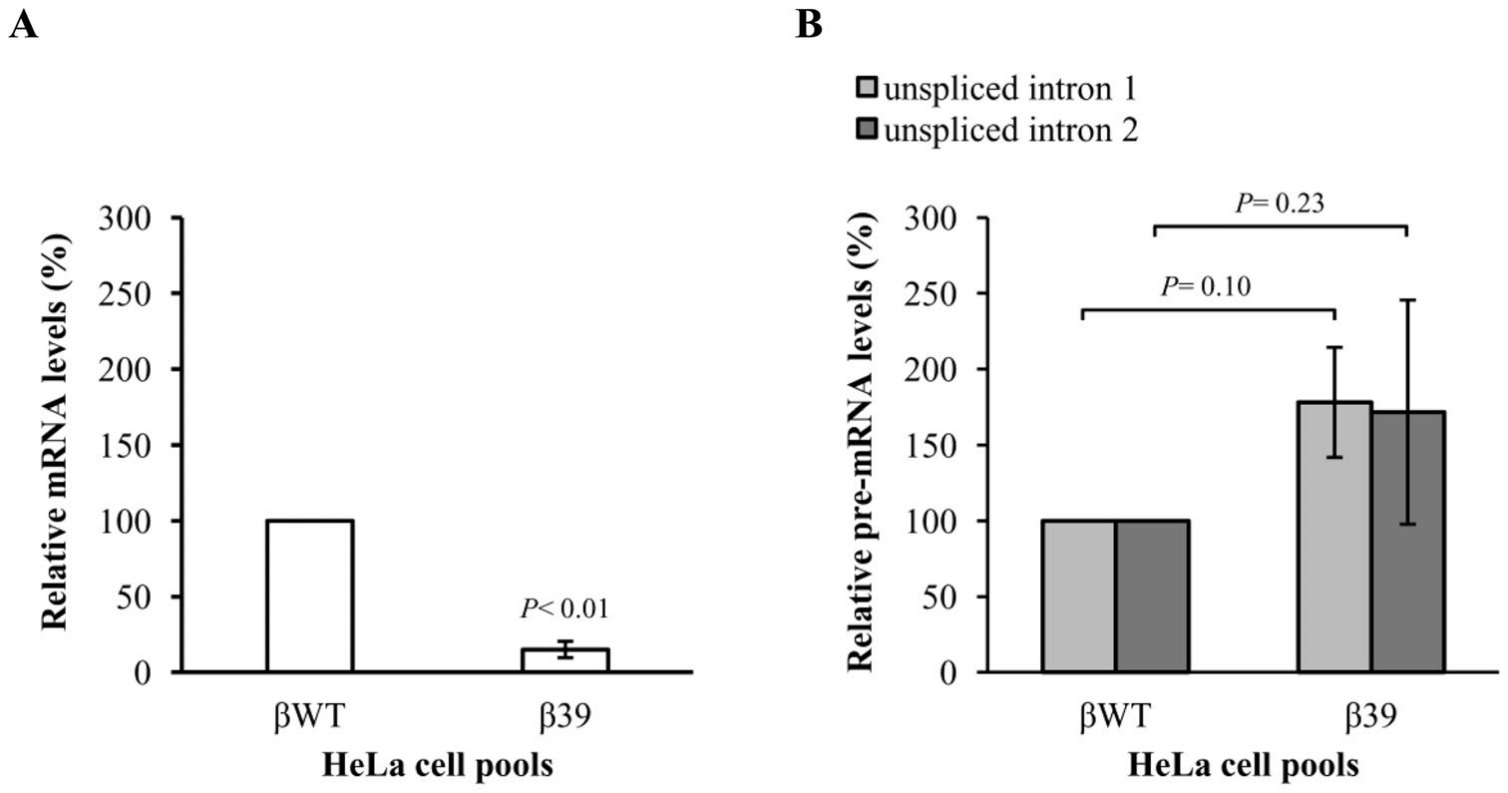
The nonsense codon effect on the β-globin pre-mRNA abundance exhibits cell line specificity. (A) HeLa cells were stably transfected with the βWT or β39 constructs as indicated below the histogram. Total RNA was isolated and βWT and β39 steady-state mRNA levels were quantified by RT-qPCR using specific primers for the human β-globin processed mRNA, as in Figure 5C, D. The histogram shows the mean and standard deviations from three independent experiments. Statistical analysis was performed using Student's *t* test (unpaired, two-tailed). (B) Total RNA was also analysed by reverse transcription-coupled quantitative PCR (RT-qPCR), with specific primers for the human β-globin pre-mRNA, as in Figure 5A, B. For each case, intron 1 and intron 2 containing human β-globin pre-RNA levels were determined by normalization to the level of the puromycin resistance mRNA, using the comparative Ct method, and compared to the wild-type control. The percentage pre-mRNA values were plotted for each construct and the histogram shows the mean and standard deviations from three independent experiments. Statistical analysis was performed as in (*A*).

To assess whether the reduced nonsense pre-mRNA levels phenotype is cell line-specific, we next analyzed the abundance of βWT and β39 pre-mRNAs in non-erythroid cells. Thus, HeLa cells were stably transfected with the βWT or β39 genes, which were previously cloned into the pTRE2 vector, behind the human cytomegalovirus promoter. The corresponding stably expressed spliced and unspliced human β-globin transcript levels were quantified by RT-qPCR analyses as before (Figure 7). Although the PTC-bearing β-globin mRNA steady-state level is downregulated (Figure 7A), as expected for a transcript typically committed to NMD [32]–[35], the corresponding β39 unspliced RNA steady-state level is neither lower nor significantly different relatively to the βWT control (*P*>0.05) (Figure 7B).

**Figure 8:**
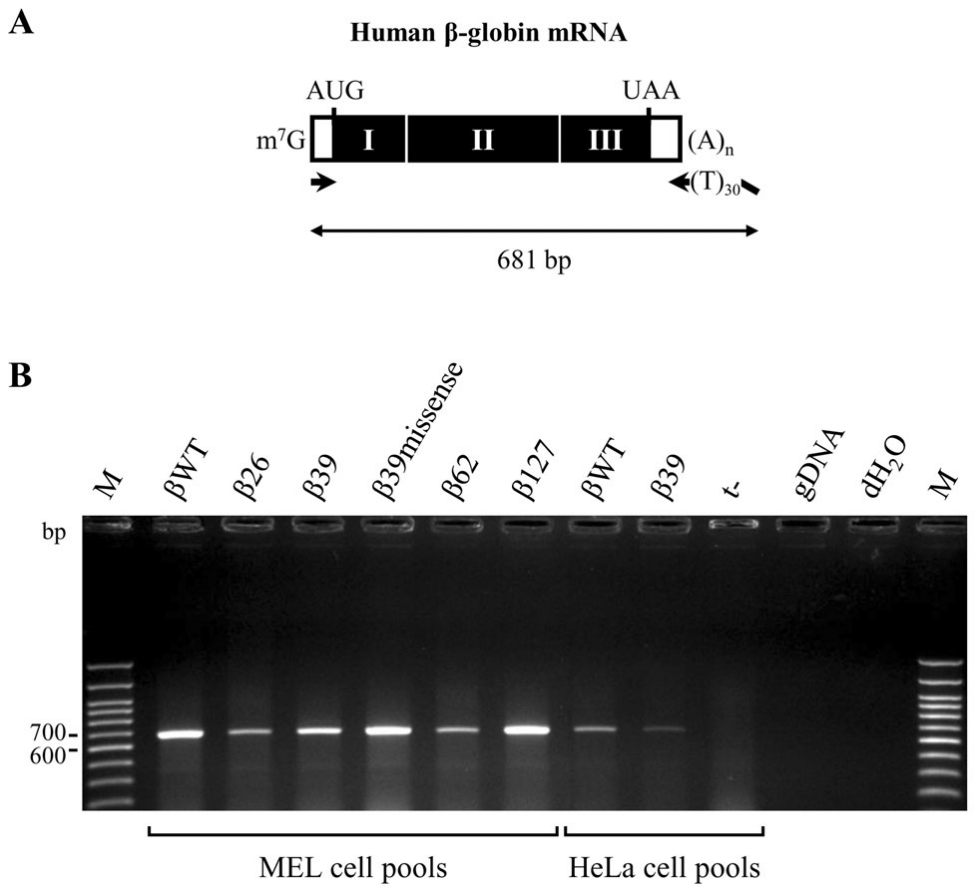
The structures of the reporter mRNAs indicate that the corresponding transcripts are normally spliced. (A) Schematic representation of the human β-globin mRNA as in Figure 2. The small arrows represent primers localization for reverse transcription and PCR reactions. Reverse primer contains a 30 nts poly(T) tail as well as a degenerated sequence. Below, the full-length of the processed mRNA is also indicated. (B) Representative ethidium bromide-stained agarose gel with the structural analysis of the human β-globin mRNAs stably expressed in MEL or HeLa cells, as indicated below the gel. The identity of each construct is indicated above the respective lane. RNA from untransfected (t-) cells, human genomic DNA (gDNA) and water (H_2_O) were used as negative controls. The molecular weight marker (M) is the 100 bp DNA ladder (Life Technologies).

Taking in consideration that nonsense mutations could introduce processing defects in the reporter nonsense transcripts that in MEL and HeLa cells would require different splicing enhancers, which would explain the different results in the two cell lines, we carried out 3′ rapid amplification of cDNA ends (3′-RACE) experiments using primers that amplify the full-length transcript (Figure 8A), thus to analyze the integrity of the transcripts. This study was conducted for all constructs expressed in MEL or HeLa cells. As expected, all cDNAs generated a product of 681 bp (Figure 8B). Furthermore, sequencing analyses of these fragments did not reveal any abnormal splicing event (data not shown). Thus, these results demonstrate normal splicing patterns for all the analyzed transcripts. Therefore, from this full set of data, we can conclude that the decreased β-globin unspliced RNA levels observed in MEL cells due to the presence of a NMD-sensitive nonsense codon is a cell line-specific effect. In addition, as reporter genes are expressed in MEL and HeLa cells under the control of different promoters, a promoter-specific effect cannot be excluded.

## Discussion

In this study, we have shown that the human β-globin pre-mRNAs carrying a NMD-competent PTC accumulate at low steady-state levels. Our results have shown that this effect depends on the presence of a NMD-sensitive PTC, although independently of its position. Functional analyses of these pre-mRNAs in MEL cells demonstrate that their low steady-state levels do not reflect significantly lower pre-mRNA stabilities when compared to the normal control. Furthermore, our results also provide evidence that, in these transcripts, the relative splicing rates of intron 1 and 2 are similar. Our results indicate that in the human β-globin transcripts, the NMD-competent nonsense codons can be recognized as abnormal during the nuclear mRNA metabolism through a promoter- and/or cell line-specific pathway. Thus, this work provides evidence that NMD-competent nonsense codons can specifically impact on nuclear regulation of the corresponding transcripts. Several nuclear RNA metabolism events could account for the decreased levels of PTC-bearing β-globin pre-mRNA, namely an abnormal rate of transcription, splicing or degradation of the nascent precursors. For instance, mammalian nuclear RNA surveillance pathways that rapidly degrade aberrant pre-mRNAs have been reported [40]. However, pre-mRNAs containing nonsense codons were never described as substrates for rapid nuclear degradation. Indeed, Lim et al [39] compared the half-life of a β-globin pre-mRNA carrying a frameshift mutation that introduces an inframe PTC between codons 60 and 61, relatively to wild-type β-globin pre-mRNA, expressed in transgenic mice erythroid cells [39]. These authors described a similar half-life for both transcripts. Our results are consistent with this observation, as we found no significant differences between the half-lives of β-globin pre-mRNA bearing a PTC at codon 39 and the normal β-globin pre-mRNA, expressed in stably transfected MEL cells. On the other hand, transcripts with processing defects are the most common substrates for nuclear RNA quality control [41], inefficient splicing being a major cause for decay [41]–[45]. This evidence directs us to the second possible explanation for the observed decreased steady-state levels of the β-globin pre-mRNAs carrying a NMD-sensitive PTC: the presence of the nonsense codon has an effect on pre-mRNA splicing. Some studies have suggested that PTCs can affect the splicing process directly, either by inhibiting splicing or by regulating splice site selection [17], [19], [46]. However, in many cases, these effects may result from the disruption of an exonic splicing enhancer (ESE) by the mutation that also generates the nonsense codon [47]–[49]. For instance, in opposition to our results, Mühlemann et al (2001) observed that PTCs in the T cell receptor-β and immunoglobulin-μ genes cause not lower but higher levels of unspliced precursor mRNAs [29]. This described nonsense-mediated upregulation of pre-mRNA was later attributed to other factors not involving recognition of a PTC, namely the disruption of ESEs [49]. Moreover, several studies conducted in β-globin, triosephosphate isomerase, adenine phosphoribosyltransferase or immunoglobulin-μ genes did not find differences in the splicing or polyadenylation events in transcripts bearing nonsense codons comparatively to the wild-type [15], [16], [18], [39], [50], [51]. Moreover, neither Maquat et al (1981), Lim et al (1992), nor Inácio et al (2004) observed any abnormal splicing rate or pattern for the β-globin transcripts bearing PTCs in erythroid cells [36], [39], [50]. The results presented here are in accordance with the previous ones, as removal efficiency of intron 1 *versus* intron 2 does not seem to be affected and the structure of the processed mRNAs is normal. Thus, the nonsense mutation does not affect mRNA processing. Therefore, another possible interpretation of our data is that the reduced pre-mRNA steady-state levels of the NMD-sensitive transcripts results from impaired transcription. A number of studies examining the abundance of PTC-containing pre-mRNAs relatively to the wild-type counterparts in different genes, including β-globin, have not detected reduced steady-state levels or transcriptional alterations [14]–[16], [18], [39], [50]. In what concerns the β-globin pre-mRNA steady-state levels in erythroid cells, the sensitivity of the assays based on S1 nuclease mapping and RNA blotting could explain the discrepancy with our results.

Another aspect of this work is the promoter- and/or cell line-specificity of the reduced steady-state levels of the β-globin pre-mRNA effect, which is specific for those transcripts bearing an NMD-competent PTC. Since β-globin genes assemble into transcriptionally silent heterochromatin in HeLa cells [52], we have analysed HeLa cells stably expressing cytomegalovirus promoter-driven βWT and β39 constructs and observed no decrease of the steady-state level of pre-mRNAs bearing a NMD-sensitive PTC relatively to the βWT pre-mRNA. This finding raised the possibility that a promoter-specific effect is responsible for the β39 pre-mRNA downregulation in MEL cells, as these cells were transfected with β-globin constructs driven by their native promoters. In fact, Enssle et al (1993) demonstrated that the nature of the promoter can dictate the fate of the β-globin transcripts [53]. Nonetheless, Bühler et al (2005) analysed HeLa cells stably transfected with the βWT and β39 genes driven by the β-globin promoter, and found no evidence for transcriptional gene silencing induced by the PTC [54]. A novel mechanism has been described, which involves an unexpected transcriptional silencing of genes bearing nonsense codons. This nonsense-mediated transcriptional gene silencing (NMTGS) seems to be peculiar to immunoglobulin (Ig)-μ and Ig-γ nonsense-containing minigenes in stably transfected HeLa cells, and was shown to result from chromatin remodelling [54]. NMTGS is specifically triggered by recognition of the nonsense codon, as it is reversed by translation inhibition and the downregulation of the essential NMD factor UPF1 [55]. However, the NMTGS physiologic role still remains elusive as no difference in the levels of nonsense codon-containing and productive immunoglobulin pre-mRNAs were detected in a B cell line, at least for the analysed differentiation stage [56].

More recently, it has been shown that the regulatory effect of NMD on gene expression of many normal mRNAs is exerted in a cell type-specific and developmentally-regulated manner, which supports the idea that the NMD surveillance mechanism may have tissue-specific characteristics [57]. Specialized nuclear pathways for regulation of the NMD-competent transcripts may be superimposed on the general NMD pathway to help making it more efficient in cell types where specific transcripts are expressed at very high levels. This reality may have driven the erythroid cells to evolve very efficient and/or superimposing mechanism(s) for recognizing and degrading nonsense globin RNAs. Different sets of data are indeed in conformity with the occurrence of tissue-specific distinctive NMD features/branches. For instance, it has been reported that nonsense codons decrease the abundance of mRNAs by reducing the human β-globin mRNAs cytoplasmic half-life in erythroid cells [58], whereas the presence of a nonsense codon also reduces the nuclear β-globin mRNA half-life in non-erythroid cells [15], [52], [59], [60]. Furthermore, along with a strong downregulation of β-globin nonsense mRNAs, erythroid cells generate detectable β-globin decay intermediates [39], [58], [61], [62], possibly resulting from tissue-specific endo- and exonucleolytic activities that may act concomitantly with the typical degradation pathways of NMD. Moreover, a cell-type specific mRNA surveillance pathway was already described in MEL cells, named ribosome extension-mediated decay (REMD), which is dependent on translation and results in the repression of the protein synthesis from an abnormal human α-globin gene containing an anti-termination mutation [63]. As tissue-specific idiosyncrasies might not provide major contributions to the overall elucidation of the NMD mechanism, they could be crucial to understand the pathophysiology of some diseases induced by nonsense mutations. In more specialized and differentiated cells, while NMD is still holding the major role, supporting mechanisms may come into the spotlight in the RNA quality control screen for transcripts bearing nonsense codons.

In summary, we show that only those NMD-sensitive human β-globin transcripts are specifically recognized as abnormal during their nuclear metabolism, being downregulated in a promoter and/or cell line-specific manner. This set of data highlights potential specialized nuclear pathways for regulation of the NMD-competent transcripts that may collaborate with, or be superimposed to the general NMD mechanism probably to achieve optimal NMD activity. Future efforts addressing these pathways will contribute to our understanding of nuclear mRNA quality control.

## Materials and Methods

### Gene constructs

Plasmids containing the human β-globin gene were derived from p158.2 [32], which comprises a 4.1 kb genomic fragment encoding the entire gene along with 0.8 kb of the 3′ flanking region and 1.7 kb of the 5′ flanking sequence comprising the promoter, adjacent to a 1.9 kb DNA fragment of the human β-globin locus control region DNase I hypersensitive site 2. Variant β-globin genes carrying the β26 (codon 26 GAG→TAG), β39 (codon 39 CAG→TAG), β62 (codon 62 GCT→TAG) or β127 (codon 127 CAG→TAG) mutations were obtained as previously described [32], [36]. The β39missense gene variant was originated from the βWT human β-globin construct by the introduction of a CAG→GAG missense mutation at codon 39 *via* site-directed mutagenesis, using the QuikChange Site-Directed Mutagenesis Kit (Agilent Technologies) with the specific primers 5′-GGT CTA CCC TTG GAC CGA GAG GTT CTT TGA GTC-3′ and 5′-GAC TCA AAG AAC CTC TCG GTC CAA GGG TAG ACC-3′. The pTRE2pur vectors (Clontech) encoding the wild-type or the β39 genes under the control of a cytomegalovirus promoter and a puromycin resistance gene were cloned as described by Silva et al (2006) [64].

### Cell culture, stable transfection and drug treatments

Mouse erythroleukemia (MEL) C88 cells [65] were cultured in RPMI medium with glutamax (Life Technologies), supplemented with 10% (v/v) fetal bovine serum at 37°C and 5% CO_2_. Stable transfection of MEL cells was carried out by us as previously described [36], using 50 μg of linearized p158.2-βWT or its derivatives, mixed with 2 μg of linearized pGKpuro, to obtain βWT, β26, β39, β39missense, β62 or β127 cell lines. Each cell pool was expanded in selective medium by adding 2.5 μg/mL puromycin (Sigma-Aldrich) and single-cell clones were established by the limiting dilution method. Erythroid differentiation was induced in equal amounts of transfected MEL cells by adding 2% (v/v) dimethyl sulfoxide (DMSO) to the media during 4 days. For pre-mRNA half-life determination experiments, the transcription of reporter genes was inhibited by addition of actinomycin D (Sigma-Aldrich) to a final concentration of 5 μg/mL, after induction of erythroid differentiation during four days. RNA was isolated 0, 15 and 30 min after transcription arrest.

HeLa cells (ATCC CCL-2) were grown in DMEM medium (Life Technologies) supplemented with 10% (v/v) fetal bovine serum. Stable transfection with the pTRE2pur-βWT or pTRE2pur-β39 plasmids and subsequent cell selection with puromycin were performed as previously described [66].

### Copy number analysis

The structure of the transgene in each MEL cell clone was determined by Southern blotting of genomic DNA from transfected MEL cell lines, isolated by the standard phenol:chloroform method and digested with EcoNI and KpnI. Digested DNAs were agarose gel-fractioned and transferred by Southern blotting onto Hybond N+ membranes (GE Healthcare). Blots were hybridized with a human β-globin gene probe labeled by the Multiprime DNA Labeling Kit (Amersham) using [α-^32^P]dCTP. This probe consists of a 768 bp EcoRI-PstI human β-globin gene fragment. Hybridization reactions, washing and exposure were carried out following the manufacturer's instructions (GE Healthcare).

To determine the transgene copy number of MEL cell clones, the human β-globin transgene copy number was compared with that of an endogenous diploid reference, the murine thymus cell antigen 1 gene (Thy1; MGI: 98747), by real-time PCR, performed in an ABI Prism 7000 Sequence Detection System, using SYBR Green Master Mix (Life Technologies). Quantification was performed by the relative standard curve method, using serial dilutions of a plasmid carrying one copy of β-globin and Thy1 gene sequences. The forward and reverse primers for the β-globin gene were 5′-GATCTGTCCACTCCTGATGC-3′ and 5′-AGCTTGTCACAGTGCAGCTC-3′; for the Thy1 gene, primers were 5′-GGTCAAGTGTGGCGGCATA-3′ and 5′-GAAATGAAGTCCAGGGCTTGG-3′.

### RNA isolation

Total RNA from MEL and HeLa cells was extracted using the RNeasy Total Kit (Qiagen) following the manufacturer's instructions. RNA samples were treated with RNase-free DNase I (Life Technologies) and purified by phenol:chloroform extraction.

### Ribonuclease protection assays (RPAs)

The used RPA probes were generated by *in vitro* transcription of plasmids containing DNA fragments from human β-globin intron 1 and exon 2 [67], β-globin intron 2, β-globin intron 2 and exon 3 [67], murine α-globin intron 1 and exon 2 (Hba-a1, MGI: 96015) [68] or murine GAPDH (MGI: 95640; pTRI-GAPDH, Life Technologies). The βintron2 probe is a 352 bp PCR-generated fragment comprising nucleotides 464 to 815 of the β-globin intron 2, which was inserted into the cloning site of pCR2.1-TOPO (Life Technologies). Each transcription vector was linearized and transcribed in the presence of [α–^32^P]CTP (Perkin Elmer) using a Maxiscript T7/SP6 Kit (Life Technologies) under standard conditions. Ribonuclease protection assays were performed using 5 to 12 μg of total RNA as previously described [36]. Radioactivity in bands of interest was quantified by phosphorimaging, using a Typhoon® Imager 8600 (GE Healthcare). The human β-globin pre-mRNA and mRNA hybridization signals from the MEL cell clones and pools were normalized to the respective endogenous control mRNA signal and compared with the reference βWT counterparts. In MEL clones, β-globin expression levels were also normalized to the transgene copy number.

### Reverse transcription-coupled quantitative PCR (RT-qPCR)

First-strand cDNA was synthesized from 1 μg of total RNA using the SuperScript II Reverse Transcriptase (Life Technologies) according to the manufacturer's instructions. From all cDNA samples of MEL and HeLa cell pools, a single full-length product was amplified using specific primers for the human β-globin 5′ and 3′ untranslated regions and then sequenced. Real-Time PCR was performed with the ABI Prism 7000 Sequence Detection System (Life Technologies) using SYBR Green Master Mix (Life Technologies). The relative expression levels of the β-globin mRNA and pre-mRNA were normalized to the endogenous GAPDH mRNA in MEL cells, or to the internal control puromycin resistance mRNA in HeLa cells, and calculated using the comparative C_t_ method (2^−ΔΔCt^) [69]. The C_t_ values of variant β-globin mRNA and pre-mRNA amplicons were compared to the respective βWT counterpart and normalized with the reference amplicon Ct value. The amplification efficiencies of the β-globin target and the GAPDH or puromycin reference amplicons were determined for each assay by dilution series. The forward and reverse primers for the human β-globin mRNA were 5′-GTGGATCCTGAGAACTTCAGGCT-3′ and 5′-CAGCACACAGACCAGCACGT; for β-globin intron 1 pre-mRNA were 5′- GCACTGACTCTCTCTGCCTATTGGT-3′ and 5′- GGGTTGCCCATAACAGCATCAGGA-3′; and for β-globin intron 2 pre-mRNA were 5′-CTGGCTCACCTGGACAACCTCAAGG-3′ and 5′-AGCGTCCCATAGACTCACCCT-3′. The primers for the murine GAPDH mRNA were 5′-ATCACCATCTTCCAGGAGCGA-3′ and 5′-AGCCTTCTCCATGGTGGTGAA-3′, and for the puromycin resistance mRNA were 5′-CGCAACCTCCCCTTCTACG-3′ and 5′-GGTGACGGTGAAGCCGAG-3′. To check for DNA contamination, quantitative PCR without reverse transcription was also performed for all samples.

### 3′-Rapid amplification of cDNA ends (3′-RACE)

First-strand cDNA synthesis was performed on 3 μg of total RNA from each MEL and HeLa cell pool using the SMART RACE DNA Amplification kit (Clontech), according to the manufactureŕs instructions. The 3′-RACE PCR covering the entire β-globin mRNA was performed with the synthesized cDNAs using primers 5′-ACATTTGCTTCTGACACAACTG-3′ and Nested Universal Primer A Mix (Clontech). After initial denaturation for 5 min at 95°C, cDNA amplification was carried out for 28 cycles using AmpliTaq polymerase (Roche) and 1 min 95°C, 1 min 58°C, 1 min 72°C as cycling conditions. The products were subjected to electrophoresis in a 1% agarose gel.

### Statistical analysis

Results are expressed as mean ± standard deviation from at least three independent experiments. Student's two-tailed *t* test was used for estimation of statistical significance. Significance for statistical analysis was defined as a *P*<0.05.
